# Death and Dying in Camille Rosalie Claudel’s Life and Work: A Psychobiography

**DOI:** 10.5964/ejop.19297

**Published:** 2025-11-28

**Authors:** Claude-Hélène Mayer

**Affiliations:** 1 Department of Industrial Psychology and People Management, University of Johannesburg, Johannesburg, South Africa.; Department of Psychology, University of the Free State, Bloemfontein, South Africa

**Keywords:** women in arts, sculptress, France, 19^th^ and 20^th^ century, psychobiography, death studies, dying, meaningful life, symbolic death, physical death, spiritual death, psychological death

## Abstract

This article examines the topic of death and dying in the life and creative work of the French sculptress Camille Rosalie Claudel who lived from 8 December 1864 to 19 October 1943. Claudel was a talented artist and sculptress who learned from and worked with Alfred Boucher and Auguste Rodin. Rodin became her professional and intimate partner for more than 10 years. Claudel was one of the few women sculptresses of the 19^th^ century to gain a reputation for the originality and quality of her work. The aim of the article is to explore death and dying in the life of Claudel. This psychobiography uses four theoretical approaches, namely existential psychology theories on death and dying, symbolic death theory, Lacanian death theory and Jung's perspectives on death, dying and *ars moriendi.* The research methodology applied is psychobiography within a hermeneutic–interpretivist research paradigm. The subject of research, Camille Rosalie Claudel, was purposefully chosen. Findings show that Claudel expressed her inner life and journey, her search for meaning, her artistic talent and in-depth philosophical interest, but also her views on life and death, through her art. As a person, she experienced different forms of death and dying throughout her life which are outlined in this psychobiography based on the four introduced theories. Conclusions are drawn and recommendations are given.

*To die is poignantly bitter,*

*but the idea of having to die*

*without having lived*

*is unbearable.*

*– Erich Fromm*

## Women Artists in Historic France

The perception of the artist as a talented, gifted and unique individual developed in the *Renaissance* in Europe during the 15^th^ to 17^th^ centuries (Hauser, 1951; Vale, 2020; Vohra, 2025). From this time onwards, artists were influenced by the rise of the merchant classes in France and Italy and by humanist philosophies and ideas (Shiner, 2001; Zhang, 2024). During this period, France became the centre of the cultural and artistic movement. It was associated with the pan-European *Renaissance* and the cultural and artistic “rebirth” of Europe (Michelet, 2010). New artistic developments in printing, painting, sculpturing and architecture occurred at that time, together with the elaboration of new codes of etiquette, sociability and original humanist discourses (Vale, 2020). However, the arts were dominated by men and women artists were hardly accepted by society (Wilson, 2010). This resulted in the situation that only very few arts institution provided entry for women artists.

In the 19^th^ century, France was influenced by the antagonism of republicans and anti-republicans, the fight of the republic against the Catholic church, by the divisions of the urban working class, bourgeoisie and peasantry, and by gender inequalities and ethnic conflicts (Delamaire & Slauter, 2021; Fox, 2012; Strikwerda, 2024). In the first half of the 20^th^ century, the period of occupation during World War I, the Great Depression of the 1920s and the defeat of 1940 during World War II all strongly affected France (Golan, 1995; Hewitt, 2003). The country became a very powerful European nation in which arts and politics were often intertwined (Fox, 2012; Gerson, 2003; Smith, 1998).

Women artists have often been underrepresented and neglected in European art history, especially in the 19^th^ and 20^th^ centuries (Wolff, 1981). Only recently have they been given the space they deserve in the arts (Grindley, 2024; Hemmer, 2024; Mahon, 2011). Thousands of women were “committed to French psychiatric hospitals between 1854 and 1943” and very few experiences have been documented (Wilson, 2010, p. 2). This happened based on the integration of “alienist medicine” and new approaches to psychiatry (around 1842) in the country (Wilson, 2010, p. 18). Women were committed in accordance with a new law. Either a relative could commit them based on declared mental illness which had to be supported by the diagnoses of two medical doctors — which was called “voluntary confinement” (Riefe, 2024), or, in the case of a public authoritarian announcement, mainly through the police, doctors had to establish sanity or insanity within 14 days (Wilson, 2010). In both cases, the person committed could only leave when family members agreed that they had been cured, or when doctors had declared them cured and sane. There was a strong debate regarding false commitment of patients in asylums, and concern about psychiatrist practices and power (Rigoli, 2001), however, the number of women incarcerated in psychiatric asylums increased dramatically towards the end of the 19th century (Ripa, 1986).

## Camille Rosalie Claudel

Camille Rosalie Claudel was born in 1864 in Fère-en-Tardenois, Aisne in northern France (Phaidon Editors & Morrill, 2019). Her parents were Louis-Prosper Claudel and Louise-Athanaïse Cécile Cerveaux and she was the second-born child in the family. Her older brother died soon after his birth. Camille, who was born only a year after her brother's death, received the name Camille which is a male and female at the same time (Chapman, 2012). Claudel (1864–1943) was followed by a younger sister, Loise Jeanne Elizabeth (1866–1935), and a brother, Paul (1868–1955). Claudel´s mother detested her for not being a boy, did not approve of her artistic involvement and expelled her from the home already early in her career as a sculptress (Runco & Pritzker, 2011). Claudel had a close relationship with her siblings, especially during her childhood years. However, the only family member supporting her throughout her life was her father (Ayral-Clause, 2002).

Claudel was educated by the “Sisters of Christian Doctrine” from the age of 5 to 12 years and later studied at the *Académie Colarossi*, one of the very few French arts school that took on women artists (Heller, 2000). She became a student of French sculptor Alfred Boucher from 1876, until he left for Italy in 1882. She then was introduced to Auguste Rodin, another French sculptor, who became her mentor, lover and colleague in 1883 (Volpi, 2025). She ended the relationship in 1892 after an abortion in connection with the fact that Rodin did not want to end his long-term relationship with Rose Beuret (Attewell, 2022; Callahan, 2015).

Claudel then mainly worked for herself for the next 20 years from 1893–1913 (TheArtStory, 2025). However, after the end of her relationship with Rodin, Claudel’s mental and physical health declined until she was “voluntarily confined” in 1913 by her mother and brother, shortly after the death of her father (Lhermitte & Allilaire, 1984; Musée Rodin, 2025). She stayed for the rest of her life incarcerated and died in 1943.

The letters and communication between Claudel, her family and friends during her long years of incarceration offer one of the few voices of women artists in France at that time (Caranfa, 1999; Rivière & Gaudichon, 2003; Wilson, 2010). They were only published in the 1980s (Rivière & Gaudichon, 2003) and provide an insight into the “artistic stasis of her mental illness and the restrictions of the asylum regime” (Wilson, 2010, p. 1). They demonstrate how Claudel resisted the “reductive process of contemporary psychiatric diagnosis” (Wilson, 2010, p.2) and show unique ways of interpreting her life.

## Aims and Research Question

This article explores the life and death of the French artist and sculptress, Camille Claudel, and focuses particularly on the themes of death and dying in her life and work, in accordance with this Special Issue which pays tribute to the study of “Death and Dying in Psychobiography”.

It is hypothesised that Claudel experienced many forms of death and dying throughout her life and used her art to overcome them. Some of her art pieces even reflect the theme of death, too (Caranfa, 1999; Claudel, 1905; Paris, 1988; Paris & de la Chapelle, 1990). She physically died in relative obscurity and only later gained recognition for her figurative bronze and marble works and her innovative and emotionally expressive style (Claudel, 1951; Krause, 2014), making her life and work infinite.

This psychobiography contributes to previous psychobiographies on artists, exploration of the life and works of extraordinary French individuals, psychobiographies of French women artists of the 19^th^ and 20^th^ centuries, and finally, to death research through psychobiography.

The main aim of this article is to explore death and dying in the life of Claudel through the lens of selected theoretical approaches. In so doing, the researcher responds to the overall research question: *How are death and dying represented in Camille Rosalie Claudel’s life and work?*

## Psychobiographical Studies

Psychobiography has a very vivid and significant history in the European context (Mayer et al., 2021a; Ponterotto, 2015a). The European Psychoanalytical Foundation was founded in 1966 and endorsed by the Psychoanalytical Association in 1969, contributing to psychoanalysis including psychobiography. Developments on the European continent in the 20^th^ century contributed to establishing psychobiography as a method which included multi-theoretical models, psychoanalysis, psychodynamics and personology (Ponterotto, 2015a). Citlak (2021) notes that psychobiography has been published not only in the Western European tradition, but also in the Eastern European tradition of the sciences, particularly in the Lvov-Warsaw School and in the psychological tradition of Twardowski’s school.

Early psychobiographical works on artists include Vasari’s (1998) study of the life of artists published in 1550 and Freud’s (1957) study of Leonardo da Vinci. Previous psychobiographies on French historical and contemporary individuals exist (Orr, 1996), including, for example, the French-Peruvian feminist Flora Tristan (Lodi-Smith et al., 2023), existential philosophers such as Albert Camus (Mayer, 2021) and Jean-Paul Sartre (Mayer & Bahrs, 2025), Gustave Flaubert (Titiriga, 2024) and psychobiographies on the modern French court (Marvick, 1996) and Cardinal Richelieu (Marvick, 1983). This study builds on expanding previous studies focusing on artists by contributing to: 1. psychobiographical French women artist studies, 2. psychobiographies in French historical contexts, and 3. psychobiographical research that focuses on death and dying.

## Selected Theoretical Approaches to Death and Dying

In the following, selected theoretical approaches are presented which build the foundation of exploring and understanding death and dying in Claudel’s life in depth.

### Existential Psychology on Death

In existential psychology, death is an important part of life which helps individuals to self-actualise and develop consciousness (Adams, 2010; Existential Therapy, 2004; Kokosalakis, 2020; Sartre, 1956). It is assumed that humans can only live fully when they face their own mortality, and that understanding death anxiety can provide the opportunity for a meaningful authentic existence (Heidegger, 1962, 2008). Kierkegaard (2009) points out that the potential of life’s enjoyment lies in the realisation that everything must come to an end and an authentic life can only be lived through the realisation of embracing life and death at the same time. This realisation of death has two layers: There is a subconscious level of death awareness where an individual feels the anxiety of death, but chooses to rather avoid it, and there is the more conscious level of death anxiety where an individual chooses to address it and consequently becomes more aware (Hoelterhoff, 2015).

The existentialist psychologist Yalom (1980) observes that individuals who deny death often try to either become the “ultimate rescuer” or they try to “become special”. Both attempts are heroic in the sense that individuals become heroes and thereby overcome death. However, Tillich (1952) contends that to be fully alive, the reality of non-being needs to be faced. Individuals often deny death by avoiding the topic or by not living their life to the fullest (Wong & Mayer, 2023). Also, the experience of anxiety of rejection in love relationships might be a sign of death avoidance. Death can only be accepted peacefully when death is being faced (Menzies & Menzies, 2024). However, facing death also means losing control and accepting our limited knowledge. It is responded to differently across cultures (Gire, 2014).

In existential thought, the symbolic side of death is highly important, and the idea of what death means for life and for humankind is consciously affected by death and destruction (Heidegger, 2014). Death, for Heidegger, occurs as a phenomenon at the end of life, but it is also part of existence; therefore, it is “at work” throughout human life and is an active part of life (Heidegger, 2014). Shariatinia (2015, p. 96) points out that death, in Heidegger’s view, is not the end of life, but “rather ontology that can be dead” and that throughout their existence, individuals communicate with death. Death becomes a “special feature” during life and is experienced in terms of the death of the “I” rather than as an end-of-life event.

If an individual does not lead a fulfilled life, death occurs through the impossibility of leading a genuine life while being a “place of certainty” (Shariatinia, 2015, p. 96). According to Heidegger (2014), death allows existence to achieve integrity as well as an awareness that everything in the world is possible. Thereby, death provides freedom since the individual owns the possibility of dying at any moment. As soon as humans project their lives “onto the horizon of death” and are in the “being” (German: *Dasein*) — in particular in “being-towards-death” (German: *Dasein im Tod*) — humans can live their lives authentically and be liberated (Critchley, 2009; Peach, 2000).

The concept of “being-towards-death” includes four basic principles:

1. Death is non-relational (everyone has to go through death alone and cut off from relationships when dying).
2. Humans certainly die and death cannot be avoided.
3. Death is indefinite in the sense that humans do not know when it comes.
4. Death cannot be outstripped, meaning that death is the “possibility of impossibility” and represents the limit of potentiality-for-being, in which death is the condition for free action during life.

Critchley (2009) concludes: “It is only in relation to being-towards-death that I become passionately aware of my freedom”. Heidegger offers a phenomenology of a relationship with death that may positively influence conducting an authentic life (Peach, 2000). According to Marseille (2021, pp. 4–5), authenticity is connected to life and creation within a finite life:

“Authenticity relates to two important concepts, freedom and facticity. Freedom, from the existentialist view, is the reality that one’s life is boundless with possibilities to create and recreate themselves into whatever they see fit. In relation to authenticity, it is the idea that one has to “create oneself” and live in accordance with this self”.

### Symbolic Death

When individuals live genuinely and authentically, they need to accept the limitation of life, which may also represent death symbolically (Bowker, 1991; Byock, 2002; Kearl, 1989; Marseille, 2021). The knowledge of the limitedness of life is powerful; it makes individuals treasure their loved ones and provides them with an engagement with life and living on a more profound level. Death can be both a distraction from truly living and a treasure to value life more.

Schreiner (2015) differentiates between physical death that cannot be avoided and symbolic death: “Symbolic death is the death of who they really are and who they are capable of becoming. It’s a death that can be avoided by making the correct choices”. Dying is in this sense a social symbolic process (Vernon, 1974), whereby life and death are viewed as less “linear than we might like it to be”, and where time, past, present, reality and imagination blur (Robb, 2025). In symbolic interactionism, the interpretation of dying is constructed between the individual and the society (Forte, 2007) and is not only a physical reality.

The symbolic death can be viewed as a period of decline or growth, depending on the choices of the individual (Schreiner, 2015). The ideal is that a symbolic death situation may be used to live life to the fullest and in harmony with authenticity, becoming a true self. However, individuals may remain in situations that are dysfunctional or they choose stagnation over growth due to existential anxiety. This existential anxiety controls the boundaries of the comfort zone (Temple & Gall, 2018). Symbolic death is experienced when individuals stay within their comfort zone and when they do not extend themselves to be who they are or want to be. However, life transitions and crises can also be experienced as death when certain relationships, situations or events come to an end (Brammer, 1992). Symbolic death marks a radical change in one’s life, and happens when individuals exit from one’s life, or when one experiences endings of any kind (Seale, 1998). Dying is viewed as a process which often goes along with the outburst of emotions, gradual maturation, family processes, a decrease in independence and an inner experience and transformation of perception (Renz et al., 2013).

### Lacanian Death

Jacques Lacan (1901–1981), a French psychoanalyst and psychiatrist, suggests that human experiences are structured at three levels: the symbolic, the imaginary, and the real (Chiesa, 2007). The individual is first defined through language and the individual’s development depends on a network of symbolic law and language (Kim, 2015). The individual is defined by the symbolic, which represents the unconscious. The unconscious is assumed to be structured in the way that language is structured. It provides insight into beliefs, feelings and knowledge (Flusberg et al., 2024). According to Lacan (2006), there is a gap between the word and the symbolic, which needs to be understood to recognise the underlying meaning of language which is also always connected to unconscious content. Even when an individual dies, the individual stays in the Other’s fantasy as a symbolic object (Chiesa, 2007). Symbolic death marks a transition period in a lifetime and is understood in the Lacanian sense to include the individual who is born, develops and dies. Diserholt (2014, p. iii) points out that death plays an underlying role in Lacan's theory and life:

“Firstly, as meaning and language are introduced in life, life and death become inextricably bound to each other. Secondly, as long as a symbol acts as a mediator between a subject and death, making sense of death becomes an impossibility; any attempt to put death into reason, finite knowledge or generalisations fails. Finally, death as standing both inside and outside our constructed reality sheds light on a necessary relationship to death that moulds an impossible, interdependent subject”.

### Jung on Death and Ars Moriendi

Throughout his entire life, Jung was fascinated by reflections on death and the *ars moriendi* (art of dying), explored the topic from multiple perspectives and defined death as psychologically as important as birth (Jung, 1957). He viewed death as integral to life (Jung, 1959) and highlighted that through individuation, individuals come closer to death and might develop a clearer understanding of it (Jung, 1957, 1959, 1960; Jungian Center, 2025).

Jung suggests that individuals die in the way they should have lived (Jungian Center, 2025). He further argues that individuals should encounter life by living as if they were dying. Individuals need to be able to face death with equanimity, as the goal the psyche is aiming for, as an integral part of one's existence (CAJS, 2024). He believed that the psyche might exist beyond death since he assumed that it is not confined to space and time and that death is a bridge between the earthly life and the spiritual world (Jung, 1959; Jung Society of Utah, 2025). At the same time, death is part of the individuation process, and part of constructing meaning in life. However, Jung (2009, p. 267) speaks not only of outer death, but also of an inner death that might be more challenging than the outer death:

“The moon is dead. Your soul went to the moon, to the preserver of souls. Thus the soul moved toward death. I went into the inner death and saw that outer dying is better than inner death. And I decided to die outside and to live within. For that reason I turned away and sought the place of the inner life”.

Jung (1989) declares death as an archetype that is part of life and thereby makes it a whole. Based on that assumption, he aimed to accept life and death and to make death a “goal of the second half of life” (Heyning, 2021). According to Samuels et al. (2005), Jungian analysts emphasise the importance of symbols and images of death and their significance and meaning for life. At the same time, they emphasise that experiences and intimations of life need to be construed as leading towards death. Life and death are then viewed as companions, and they become equal parts of a complete human existence. Death awareness is part of individuation and helps individuals to become increasingly aware and conscious. Therefore, death needs to be viewed as a goal and the destination of an individual journey that begins long before death arrives (Purrington, 2020).

The four eclectically presented theoretical approaches on death and dying will be used to interpret Claudel’s life and parts of her work.

## Research Methodology

Psychobiography, as a subdivision of psychohistory, has a long-standing research tradition and aims at integrating biographical life narrations of a selected subject (Ponterotto, 2017a, 2017b, 2025; Schultz, 2005; van Niekerk et al., 2019), here Camille Rosalie Claudel, with psychological theory and the socio-historical context (Mayer, 2017). In the case of this psychobiography, the author focuses on theoretical aspects of death and dying from the perspectives of existential psychology, symbolic death, Lacanian and Jungian theoretical approaches. Furthermore, the subject, being a woman artist, is understood through the embedding interpretation of the subject’s life in 19^th^ and 20^th^ century France (Hemmer, 2024; Wilson, 2010; Wolff, 1981).

### Research Design

This psychobiographical account draws on a qualitative single case study design (Alexander, 1988; Carlson, 1988; Elms, 1994; McAdams, 2006; Nel et al., 2024; Yin, 2018), is highly qualitative in nature and uses a life history approach (Fouché, 2015; Fouché & van Niekerk, 2005, 2010). As a psychobiography, it uses psychological concepts to increase the understanding of the life and the concept of death of the selected individual during her lifetime (Schultz, 2014).

### Sample and Sampling

The researcher selected the psychobiographical subject purposefully (Burnell et al., 2019; Mayer, 2017). A non-probability, purposeful and theoretical sampling method was used (Kőváry, 2011), based on the judgement of the researcher to study the desired topic of a subject throughout her lifetime in a specific context, approaching the aspect of death and dying.

The researcher chose Claudel based on a number of considerations. First, Claudel was one of the most significant extraordinary female artists of 19^th^ and 20^th^ century France (Hemmer, 2024) and to date, no psychobiography had been done on her. Second, she was affected by different deaths in her life and she lost her career and life of freedom, facing a symbolic and social death in the France of her time since she was an extraordinary woman artist who did not conform to societal norms. Finally, she spent several decades in an asylum (Krause, 2014), facing an isolated life and the death of her artistic identity.

The researcher identified Claudel as a significant individual in the context of death studies, and in relation to the researcher’s own interests (Gardner, 1999; Howe, 1997). The researcher was particularly interested in France, French historical and contemporary philosophy and arts (Mayer, 2021; Mayer & Bahrs, 2025), in exploring the lives of extraordinary women (Mayer et al., 2021b), and in analysing the life and work of the subject regarding death and dying.

The researcher first came across Claudel’s life in the 1990s when visiting Paris on a regular basis. One day, while exploring the work of August Rodin in the Rodin museum in Paris, the researcher was drawn to the life of Claudel and started to buy and read books on her life and art. Claudel’s extraordinary passion, suffering, her approach to emotions, and her resistance to French society’s norms stood out significantly. When thinking of a subject who was affected by death and dying, the first who came to the researcher’s mind was Claudel. According to the researcher’s understanding, Claudel died many times throughout her life, painful and infinite deaths. The idea underlying this psychobiography was to analyse death and dying in the subject’s life and to explore the deaths she had to face during her lifetime.

### Data Collection and Analysis

Data on Claudel were collected over a period of many years since the author has a vivid interest in philosophers, writers and artists of the 20^th^ century in France (Mayer, 2021; Mayer & Bahrs, 2025; Mayer & Gonot-Schoupinsky, 2024). Literature research was conducted, including Google Scholar, Nexus, PsychLit, and Ebscohost, to find existing psychobiographies, biographies, articles, videos, books and book chapters on Claudel’s life. Primary and secondary sources based on the subject’s life were used (Yin, 2018) to explore the topic of death and dying in the context of her life. The sources chosen to understand the life of Claudel in depth were informed by a quantity of information on her life, her artistic work and by the quality of information regarding the subject of death and dying (Mayer & Kőváry, 2019).

Primary information (Yin, 2018) included personal documents, diary entries, letters (Claudel, 1891) and secondary sources such as articles, books and book chapters, biographies and biographical notes (Attewell, 2022; Ayral-Clause, 2002; Bougault, 2020; Bouté, 1995; Burgon, 2012; Callahan, 2015; Caranfa, 1999; Cassar, 1987; Claudel, 1905, 1951; Delbée, 1982; Fabre-Pellerin, 1988, 2005; Garman, 2017; Krause, 2014; Laurent & Gaudichon, 1984; Paris, 1988), art catalogues (Paris, 1984; Bowyer & Desmas, 2025), artist websites (Hemmer, 2024) and recordings, podcasts (e.g., France Culture, 2021), a musical theatre piece (Heggie & Scheer, 2012) and videos of the subject (Arte, 2024; Dumon, 2013; Nuytten, 1988) as described by Fouché and van Niekerk (2005). Some of Claudel’s sculptures and art pieces have been taken into consideration in this article (Bowyer & Desmas, 2025; Elsen & Jamison, 2003), since several of them were described as autobiographical and deal with meaning in life, philosophical content, relationships, and death and dying. No previous psychobiographies on Claudel were found during the literature review.

Data about Claudel’s life and work are extensive. The researcher therefore demarcated significant findings regarding her life and death (Alexander, 1990), guided by the study’s aim and the defined theoretical approaches. Alexander’s (1988, 1990) indicators of salience were used to question the dataset and to identify extraordinary themes. Thematic indicators include: 1. Primacy, 2. Frequency, 3. Uniqueness, 4. Negation, 5. Emphasis, 6. Omission, 7. Error or Distortion, 8. Isolation, and 9. Incompletion. These indicators are not explained further here, but can be explored in Alexander (1988, 1990).

### Quality Criteria, Ethics and Limitations

Rigor and trustworthiness were established as quality criteria in this study (Krefting, 1991; Lincoln & Guba, 1985) through the in-depth investigation of Claudel’s biographical, sociocultural and historical data. The researcher further used reflexivity, to understand and interpret the findings based on well-defined theory and methodology (Ponterotto, 2014). Theory and data triangulation were applied (Yin, 2018).

In terms of the ethical considerations, this psychobiography follows the principles discussed in recent psychobiographical discourses (Mayer & Kőváry, 2019; Ponterotto & Reynolds, 2019). Ethics are a central part of the psychobiographical research study and ethical considerations are outlined throughout the research process (Ponterotto & Reynolds, 2019). Only publicly available sources were used to reduce ethical risks and “postmortem privacy rights” and next-of-kin-rights were maintained (Ponterotto, 2015b; Ponterotto & Reynolds, 2019). By choosing a deceased subject, the ethical issues regarding the choice of the living subject were overcome (APA, 2025). Ethical approval was provided by the University of Johannesburg.

This study is limited by the selected theoretical and methodological approaches applied. Furthermore, the researcher’s personal background as a European woman interested in French culture and history who was born several decades after Claudel’s death in a neighbouring country might bias her own understanding of existential psychology and psychiatry, life and death and the life and challenges of women artists in 19^th^ and 20^th^ century France. Although quality criteria were applied in this study, the study might be limited by the researcher’s biases, affecting the possibility of self-reflection and of organising and analysing the data.

## Death and Dying in the Life of Camille Rosalie Claudel

In this psychobiographical study, the researcher first focuses on understanding death and dying through an existential perspective, seeing death and dying as a meaningful part of Camille Claudel’s work and life. Next, she analyses death and dying from a Lacanian viewpoint, thereafter describing death and dying from a symbolic perspective and finally, understanding death and dying in the subject’s life from a Jungian theoretical approach. The researcher thereby responds to the overall research question: *How are death and dying represented in Camille Rosalie Claudel’s life and work?*

### Existential Perspectives on Death in Camille Claudel’s Life

Camille Claudel’s life and its meaning were very much affected by her huge talent and by being a woman artist who was one of the most “daring and visionary sculptors of the late nineteenth century” (Bowyer & Desmas, 2025). During her childhood and teenage years, she loved being in nature, working with clay and sculpturing, expressing her creativity and representing a deeper connection to life and meaning (Kellert, 2005). This existential connection to life and meaning also connected Claudel with Bochner and later with Rodin, expressing her urge for life and to experience life in an intense and in-depth way. On the other hand, Claudel’s powerful connection to life may also have had a passionate correspondence with death. Claudel was strongly influenced by being born shortly after the death of her older brother, since her mother was upset that she was born as a girl and had expected Claudel to fill the void left by the death of her first child. However, Claudel was born a girl which evoked disappointment and rejection of her mother (Ayral-Clause, 2002). It might be even argued that this very early rejection by her mother represented Claudel’s first existential death (Weir, 2012). Later, the rejection of her mother even grew, since Claudel followed an artistic lifestyle against the societal norms for women (Ayral-Clause, 2002). This might be an outcome of the mother’s projection of her wish that Claude had been a boy since she lived her life, not fitting in the women’s roles in this French historic period.

Claudel’s life struggles became visible especially after the end of her relationship with Rodin (Bougault, 2020; Burgon, 2012; Obsolete Oddity, 2019) who experienced her as his greatest love, but also as his greatest competition (Ayral-Clause, 2002). The relationship’s end led her into a financial death and to despondency and despair (De Bayser, 2008), a form of psycho-spiritual crisis and death. De Bayser (2008) emphasises that Claudel knew both extremes: the pleasures and the pains of life.

Her love and her despair are both evident in her artworks. *Clotho* (1883) represents a drive away from Rodin and his sculpturing style (Elsen & Jamison, 2003; Peterson, 2023). It constitutes human destiny, expressed as an image of an elderly woman who is the antithesis of young and beautiful women, representing old age and death and the opposite of what is attractive from the male perspective (Peterson, 2023). *Clotho* is a symbol for a hopeless life and infinite death and could represent Claudel’s inner death.

Besides *Clotho* indicating a break of style and era in Claudel’s life, the artwork *The Wave* (1897–1903) also expresses a conscious style change which seems to be connected to the end of the romantic relationship with Rodin, indicating the death of their creative, meaningful relationship and their soul connection. *The Wave* is an emotional expression of transformation, power and existential being in the world.

The work *The Mature Age* (1899) shows the end of the relationship with Rodin. The sculpture consists of an old man who is taken away by an old woman representing Rose Beuret, symbolising old age, maturity and death. When Rodin saw the work, realising that it depicted their relationship’s story, he ended his financial support of Claudel and convinced the French government to do the same. The youth in this sculpture, representing Claudel, aims to rescue the man from old age and from the old woman who is trying to save him (Ayral-Clause, 2002; Souter, 2011) while realising that the end and symbolically the finitude of the relationship cannot be stopped. The work epitomises suffering regarding the relationship’s transformation and death, representing the end of their lives together, while at the same time symbolising despair and loneliness.

Symbolically, several of Claudel’s artworks represent change, transformation of emotional states and life phases, mortality, endings and death. Expressing these symbols contributed to her authentic existence, integrating in-depth life and living with its closeness to death and dying (Heidegger, 1962).

Kierkegaard (2009) argued that the potential enjoyment of life lies in realising the potential end of everything. In a way, this is evident in Claudel’s life. It appears that she lived her life of art and the relationship with Rodin intensively in all its depth and emotional power, always facing potential endings and death. Since Rodin lived a “double life” with Rose Beuret, Claudel must have been aware of the potential finitude of the relationship at any point in time. Her anger and rage might have been an expression of despair and refusal to accept the approaching death of the relationship, which then led her into an extreme paranoia from 1905 onwards (Obsolete Oddity, 2019). The despair in her relationships and symbolically her life’s end is expressed in, for example, her work *Fortune* (1900), which represents “her innovative spirit and her mastery of capturing the fleeting essence of life within the permanence of sculpture” (Artchive, 2025). The transitions and fleeting essences of life represent the loss of control (Gire, 2014) which Claudel faced through her art and which manifested later in her emotional outbursts and paranoia.

It could be argued here that the end of the relationship was unbearable for Claudel and caused intense suffering and therefore led her into a paranoid state, internally fleeing from dealing with the relationship’s death consciously and constructively. How conscious she was of the topic of death, however, is difficult to determine. During some phases of her life, death appears to be experienced unconsciously, while at the same time being part of her constant questioning of human destiny, as expressed in *Sakuntala* (1886–1889) and in her portraits of children — representing reflections of life, human destiny, eternity and death (Musée Rodin, 2025). She seemed to be dealing with life and death and existential topics between the extremes of living and dying and intense emotions, phases of creativity and expression, as described by Kierkegaard (2009).

Regarding the two layers of death discussed by Hoelterhoff (2015), Claudel seems to deal with death and death anxiety on both a subconscious and an avoidant level through her work and sculptures (e.g., *The Wave*). She also consciously expressed her despair at the finality of life’s phases and her relationship with Rodin through the destruction of her own work in 1912, and in 1913 when her father died (TheArtStory, 2025). The death of her father brought about a conscious death experience (Hoelterhoff, 2015) and led to high levels of suffering, despair, loneliness, anxiety and isolation (TheArtStory, 2025). The conscious level of death was also expressed in the work *The Abandonment* (1905).

Existentialist psychologist Yalom (1980) highlights that individuals who deny death often try to become either the “ultimate rescuer” or "special”. Both attempts are heroic in the sense that individuals become heroes and thereby overcome death. Claudel was not an ultimate rescuer but rather became special and extraordinary through her immense work. In this way, she overcame death and is alive in her artworks and in her extraordinary life story, representing her infinity as *the* sculptress of all times. She lived her life fully when facing death (as in Tillich, 1952) on ending the relationship with Rodin, and in extreme suffering on the death of her father, the death of her career as an artist, and the end of her life in freedom through “voluntary placement” (Musée Camille Claudel, 2025).

Claudel experienced and expressed strong emotions while encountering these deaths in her life. According to Menzies and Menzies (2024), death can only be accepted peacefully when faced. While in the first years of her “voluntary confinement” in the asylum, Claudel aimed despairingly to be released (TheArtStory, 2025). TheArtStory (2025) however describes her — when being visited by her old friend, Jessie Lipscomb, in 1929 in the asylum — as the “interesting shell of a once magnificent creature”, implicitly referring to her inner death in the asylum (Jung, 1957, 1959). The longer she remained there, the more she seemed to accept her imprisonment and the death of her life, relationships and art career (Burgon, 2012). Ayral-Clause (2002, p. 157) emphasises that through her work she transformed “because it welcomes light and radiates the inner dream that inspired it”. With the end of her work, she died an inner death.

**Figure 1:**
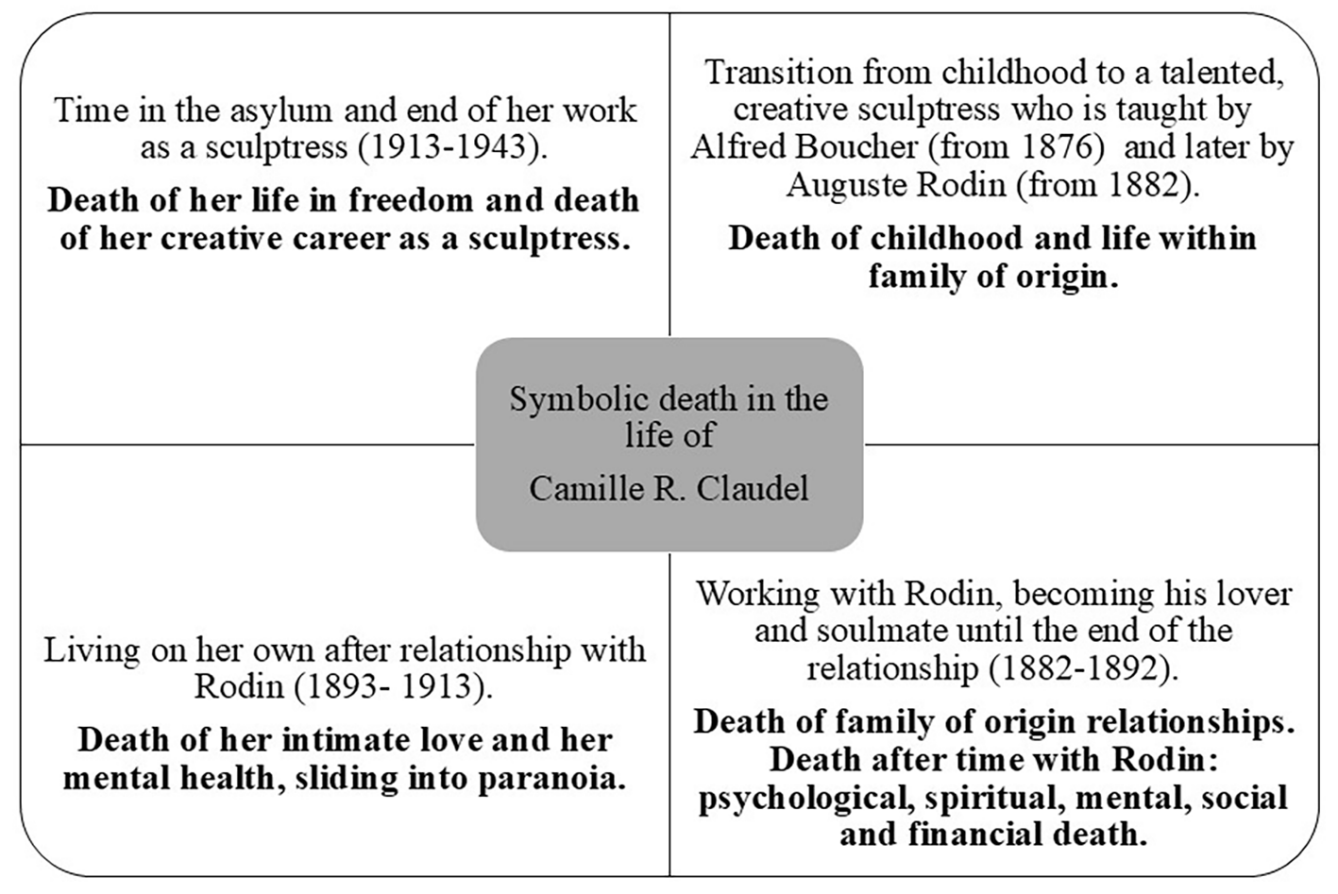
Symbolic Death in the Life of Camille Claudel

In existential thought, the symbolic aspect of death and the idea of what death means for life and for humankind is particularly important (Heidegger, 2014). Symbolic meaning in the life and works of Claudel is also significant (Burgon, 2012) and is consciously affected by death and destruction (Heidegger, 2014). Death in Claudel’s life is, as described by Heidegger (2014), not only a phenomenon at the end of life, but also part of existence; therefore, it is actively “at work” throughout human life. In Claudel’s life, death can symbolically be viewed on different layers, as shown in Figure 1.

Through her life, Claudel communicated with death, as described in Shariatinia (2015). It is hypothesised that Claudel communicated with the extremes of life (passion, love, emotional intensity) and death (destruction, paranoia, end of relationship) through her artwork, bridging the gap between the conscious and the unconscious, and beyond language in a Lacanian sense.

Especially after the end of the relationship with Rodin, death became a “special feature” for Claudel, experienced as the death of the two lovers, a death of their soul connection, a death of their common creativity and their common creative inspiration. In the end, the death of the soul connection between Rodin and Claudel led to what is described in Shariatinia (2015, p. 96) as the death of the “I”. Following a new creative wave in Claudel’s art from 1893 to 1905, her “I” became increasingly obsessed with paranoid ideas and the destruction of her own creative expression. Claudel appeared to have left the “place of certainty”, exiting her genuine, authentic and fulfilled life and experiencing psychological death (Shariatinia, 2015, p. 96). She no longer felt integrated and had clearly lost awareness of the idea that everything in the world is possible (Heidegger, 2014), retracting into a state of paranoia.

While Claudel had behaved during the ten-year relationship with Rodin as if projecting life “onto the horizon of death” and as if they were “in the being” (German: *Dasein*), they seemed to live authentically and in a liberated way (Critchley, 2009; Peach, 2000). It appears that they had lived their shared life “in the moment” and by “being towards death”. Claudel and Rodin might have aimed to overcome death (Principle 1, everyone has to go through death alone) through their love and intimate relationship. They lived their relationship to the fullest since life is finite and cannot be avoided (Principle 2) and since one never knows when it comes (Principle 3). Finally, it can be assumed that Claudel lived an existential life in which death is consciously and unconsciously may present the condition for free action during life (Principle 4). The four principles of “being-towards-death” (Heidegger, 2014) were vivid especially in Claudel’s life with Rodin. After that, life seemed to be less existential for Claudel and it seemed as if a part of her had died.

The end of the relationship with Rodin spoke to Claudel in the form of the four principles (Critchley, 2009): Through their deep love relationship, they were aware of the certainty of death and dying, the insecurity of when their end would come and their potentiality-for-being, whereby death represents the condition for free action during life. In their love relationship, they acted as if they were free of societal and other relationship bonds, acting against the societal norms (such as their age gap and Rodin’s polygamy). They lived a life of freedom and authenticity (Peach, 2000) which died after the end of their relationship. While together, it seemed as if their lives were boundless, with possibilities to create and recreate (Marseille, 2021, pp. 4–5), which was expressed in the artworks of that time.

### Symbolic Forms of Death in Camille Claudel’s Life

While overcoming death through their love relationship with Rodin (Volpi, 2025), Claudel had to accept the limitation of life through death (Bowker, 1991; Byock, 2002; Fabre-Pellerin, 1988, 2005; Kearl, 1989; Marseille, 2021), especially in accepting that Rodin would not leave his partner Rose Beuret for her. This made Claudel powerful on the one hand, treasuring Rodin’s love and their time together. However, on the other hand, it finally brought the end of their relationship, because Rodin would not decide to live a life with her exclusively.

Claudel’s physical death (Schreiner, 2015) only arrived in 1946; however, her symbolic death happened before through social-symbolic processes (Vernon, 1974) between the individual and the society (Forte, 2007). Claudel died several deaths, as shown in Figure 1.

First of all, Claudel’s death as a child and her birth into the life of becoming a talented sculptress working with Alfred Boucher, as well as her move with her family to the new town, indicated her choice of growth and development (Schreiner, 2015), as did the choice to become Rodin’s assistant and work with him for 10 years. Her move away from Rodin after this period was also a death connected to growth and development, in that Claudel entered a new phase of creativity and innovation which brought growth and development through individuation (Jung, 1959). However, it also brought death in terms of decline of her balanced life, her mental health and well-being. In this respect, she chose stagnation over growth (Temple & Gall, 2018) by choosing mental decline, paranoia and destruction over her own personal evolution and becoming an authentic person within herself without her lover and soulmate, Rodin. It might be assumed that she chose stagnation over growth due to her existential anxiety after the death of her love relationship; her stagnation kept her in the comfort zone of her own artistic life (Temple & Gall, 2018), locked up at home, without many external contacts and without going beyond her own boundaries.

The end of the relationship with Rodin was a particularly impactful death experience for Claudel, as described by Brammer (1992), which brought a transition into loneliness, mental decline, and paranoia. She experienced a process of psychological dying, showing the symptoms highlighted by Renz et al. (2013): outbursts of emotion, gradual maturation, family processes, a decrease in independence, and an inner experience and transformation of perception. The period from 1893 to 1913 symbolised the death of a certain time in her life, of the relationship that had come to an end, and of a situation that marked a radical change in her life.

Symbolic death happened to Claudel when Rodin exited her life, and when she experienced the ending of a phase of her creative work, as described in Seale (1998). She finally experienced another death when her father died. This death ended her relationship with her family, the family’s support, her acceptance by her family, and it led to her final social death when she was admitted to the asylum. This confinement brought the death of her artworks, her connection to the outside world, her family connections, and the freedom in her life.

### Lacanian Death: Claudel’s Interdependent Relationship With Death

Claudel’s life was structured as described by Lacan through the symbolic, the imaginary and the real (Chiesa, 2007). Claudel developed a network of symbolic law and language (Kim, 2015) and symbolically expressed her unconscious through her artworks. It might be argued here that life and death in Claudel are “inextricably bound to each other” through her conscious life and death as an artist on the one hand and the somewhat more unconscious expression of life and death in her artwork on the other hand (Diserholt, 2014). In her life, death has an immanent position and her artworks can be viewed as a mediator between her and her experiences of life and death. It might further be argued that Claudel had so closely bonded with death after the end of the relationship with Rodin, that her life became an “interdependent part” (Diserholt, 2014) of death, and she chose destruction and the emotional expression of existential anxiety in the form of anger and uncontrollable emotions. It could be assumed that her relationship to death became stronger than her relationship to life and therefore directed her towards loneliness, destruction and the repetitions of previous artworks rather than to creating new original work and finding new solutions to her seemingly unsolvable problems with her family, her finances, the contracts with the French government and her loneliness and despair.

### Jung on Death, Ars Moriendi and Camille Claudel’s Life

As described by Jung (1957), death became a key element in the life of Claudel and it was integral in her life and artwork. Symbols of death — as in *Crouching Woman*, *The Mature Age* or *Clotho*, for example — show the significance of age and death in her life, as described in Samuels et al. (2005). Death appeared to be something she feared already at an early age and an early career stage, dealing with it consciously.

In other artworks, such as *The Waltz*, life and death are both represented, while the death of her career and of her emotional relationship with Rodin are represented in artworks such as *The Abandonment* (1905) which shows the fractured intimacy of a man and a woman.

According to Jung (1959), individuals come closer to death through individuation, which might also have been true for Claudel. After she spent years in the asylum and had time to reflect upon her life and work, she seemed to be increasingly balanced and calm. It might be assumed that she had accepted the death of her relationship with Rodin, the death of her career and of her life as an artist. This could be interpreted — according to Jung (1957, 1959, 1960) and the Jungian Center (2025) — as her development of a clearer understanding of life and death and what it meant to her. She appears to have accepted her inner death. Claudel viewed the deaths of her relationships, her arts, her mental health and her life in freedom with equanimity. She had stopped fighting for her freedom and for a way to leave the asylum.

It might be presumed that at least twice she managed to see her life as aiming for death (as suggested in CAJS, 2024), namely at the end of the relationship with Rodin and after she had understood that she would never again leave the asylum. It could further be assumed that death, in the end, was a meaningful part of her life (Jung, 1959; Jung Society of Utah, 2025) and a goal (Heyning, 2021), since in life she was cut off from her deepest love, Rodin, from her family, her art and society. Her inner (spiritual, mental, psychological and social) death may have been much more challenging to Claudel than her physical death, as described by Jung (2009). Claudel’s soul may have moved towards death already in 1893 and she might have decided to live within herself while dying externally, wearing dirty clothes, not maintaining any contact other than with her cats, only leaving her house at night, having emotional outbursts and being placed in “voluntary confinement” (Riefe, 2024).

Through all her experienced forms of death, Claudel’s life became whole, in the sense that these extremes of living and dying led her towards great posthumous fame, not only as a woman artist, but also as a female sufferer in the male-dominated European world of 19^th^ and 20^th^ century history. She became a symbol of a heroine and female warrior of her times.

### Conclusion and Recommendations

The principal aim of this article was to explore death and dying in the life of Claudel through the lens of selected theoretical approaches. To do so, the researcher responded to the overall research question: *How are death and dying represented in Camille Rosalie Claudel’s life and work?*

Death and dying have many components in Claudel’s life and are strongly connected to her intense way of living and expressing herself in the arts, thereby creating extraordinary meaning in her life and beyond. On the one hand, **physical death** affected her life: the death of her oldest brother before her birth, the death of her cousin (1905) and her father (1913), the death of her sister (1935) and her own death in 1943. On the other hand, Claudel experienced a **financial death** after the split from Rodin (1892/1893) and later on her father’s death (1913). She also underwent what might have been the most destructive death for herself — a **psychological/mental death** after the end of the relationship with Rodin (1893) and the death of her father (1913), as well as the **death of her career** as a woman artist with her confinement in the asylum in 1913. While experiencing deteriorating mental health, Claudel also went through a **social death** since her family excluded and isolated her and she withdrew from societal life, retracting into psychological illness and paranoia.

**Figure 2:**
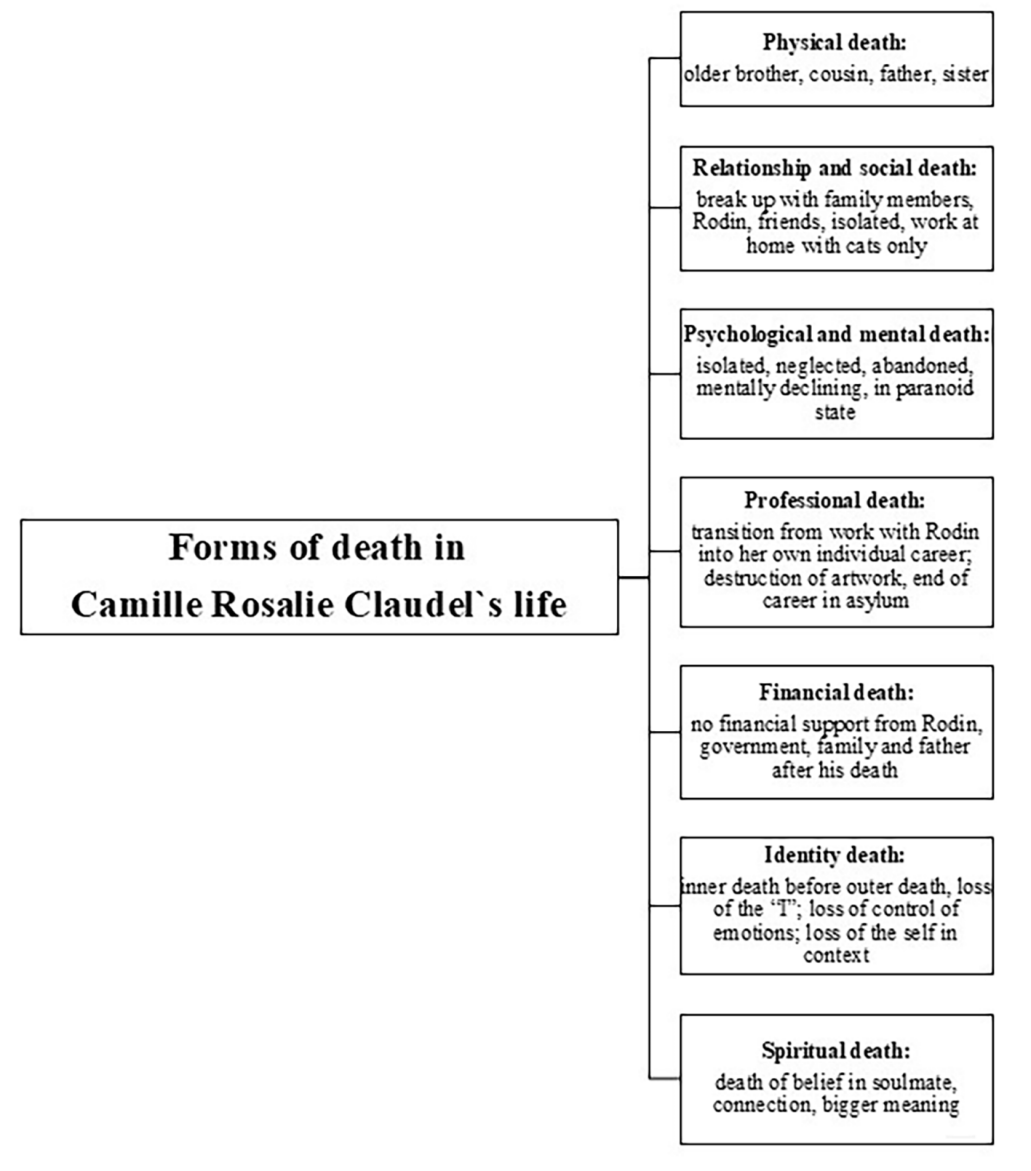
Forms of Death in Camille Claudel’s Life

In her artwork, the intensity of Claudel’s life is vivid. She lived her passion and meaning in life as an artist and Rodin’s lover intensely and experienced the **identity death** of her “I” and expressed her life and deaths in many ways. With the death of her relationship with Rodin and finally the death of her father as her only supporter in the family, Claudel’s soul connections in the world died and she experienced a **spiritual death**, which is expressed in addition to the other forms of death in various artworks. The admission to the asylum integrated all of the deaths she had died before and it can be assumed that her inner deaths were much more challenging than physical death at the end of her life (Figure 2).

This psychobiography contributes to previous psychobiographies on artists by presenting an in-depth insight into Claudel’s life with special regard to death and dying in her life and work. The study contributes further to the exploration of the life and works of extraordinary French women artists, presenting Claudel as a sculptress who became a symbol of women the many women who had been committed to asylums during the 19^th^ and 20^th^ centuries. This psychobiography also stands as an example of psychobiographies of French women artists of this period in history. Finally, the study contributes to the advancement of psychobiographical research on death and dying and to different manifestations of death from birth to physical death, drawing attention to the different ways of dying throughout life.

It is recommended that future research explores the life of Camille Claudel with regard to her relationship with Auguste Rodin, the expression of love and the consequences of unfulfilled love in her life and on her mental health and well-being. Furthermore, future psychobiographies need to take death and dying into consideration to contribute to contemporary theories of death and dying and their meaning in life and across the lifespan. Psychobiographies can be used to make death and dying a more conscious topic not only in the life of extraordinary individuals, but also in the context of contemporary societies and individuals who aim at creating meaning and impact during their lives.

On a practical note, discourses on death and dying in extraordinary individuals can inform counselling, consulting and therapy, especially those based on existential meaning therapy and perspectives using Carl Gustav Jung’s therapeutic approach. Psychobiographies could provide examples of positive and constructive ways of dealing with death and dying, to create awareness and lessons learned of meaningful ways of dealing with these existential topics.
